# Exploring the hidden synergy between system thinking and patient safety competencies among critical care nurses: a cross-sectional study

**DOI:** 10.1186/s12912-025-02717-6

**Published:** 2025-01-31

**Authors:** Amal Diab Ghanem Atalla, Rwan Ragab Rabea Bahr, Ahmed Abdelwahab Ibrahim El-Sayed

**Affiliations:** 1 Nursing Administration Department, Faculty of Nursing, Alexandria University, Alexandria, Egypt; 2 Abbas Helmy Center for Addiction and Mental Health, Alexandria Health Directorate, Alexandria, Egypt

**Keywords:** Nurses, Patient, Safety, Competencies, System thinking, Synergy

## Abstract

**Background:**

Patient safety remains a global priority, with nurses playing a crucial role in minimizing errors and improving patient outcomes. System thinking, which involves understanding how various components of a healthcare system interact, is increasingly recognized as essential for enhancing patient safety competencies.

**Aim:**

This study investigates the impact of systems thinking on patient safety competencies among nurses in critical care units, exploring the relationship between systems thinking and various subdomains of patient safety competencies.

**Methods:**

A cross-sectional study was conducted at all critical care units of Alexandria Main University Hospital, Egypt. Data were collected from a convenience sample of 289 nurses using the system thinking scale and the patient safety competency self-evaluation questionnaire. Correlation and regression analyses were performed to examine the relationship between systems thinking and patient safety competencies, controlling for demographic factors such as age, qualifications, and years of experience.

**Results:**

Nurses demonstrated moderate to high levels of systems thinking (mean = 82.36 ± 12.14) and patient safety competencies (mean = 162.74 ± 23.56). Strong positive correlations were found between systems thinking and patient safety competencies (*r* = 0.605, *p* < 0.05), particularly in areas such as error reporting, communication, and infection prevention. Regression analysis revealed that systems thinking significantly predicted patient safety competencies, increasing the explained variance from 58.8 to 67.7%.

**Conclusion:**

The findings highlight the critical role of systems thinking in enhancing nurses’ patient safety competencies. Nurses with higher systems thinking skills are better equipped to prevent errors and improve communication, ultimately enhancing patient care quality.

**Implications:**

Nursing schools should integrate system thinking into nursing curricula to prepare future nurses for complex healthcare environments. Healthcare organizations should incorporate system thinking into professional development programs to enhance the competencies of practicing nurses. Nurse managers can foster a culture of safety by promoting interdisciplinary collaboration and reflective practice. Broader adoption of system thinking can improve patient outcomes, especially in resource-constrained environments.

**Clinical trial number:**

Not applicable.

**Supplementary Information:**

The online version contains supplementary material available at 10.1186/s12912-025-02717-6.

## Introduction

Healthcare systems worldwide face significant challenges in preventing errors and ensuring patient safety. The World Health Organization reports that millions of patients globally experience preventable harm annually, resulting in significant morbidity, mortality, and financial strain [1]. In the United States, medical errors are the third leading cause of death, claiming approximately 250,000 lives each year [2]. Low- and middle-income countries face even greater challenges, with 134 million adverse events in hospitals annually causing 2.6 million deaths [3]. Egypt reflects this crisis, with studies showing that over 10% of hospital admissions are linked to medical errors, including medication and surgical complications. These statistics underscore the urgent need to enhance patient safety competencies among nurses [4].

Patient safety is a core competency for healthcare professionals, with nurses playing a pivotal role [5]. As the largest segment of the healthcare workforce, nurses spend the most time with patients, monitoring their condition, administering medication, and ensuring adherence to treatment protocols [6]. This positions them to prevent and mitigate errors effectively. However, the complexity of modern healthcare systems and the increasing acuity of patient conditions create a high-risk environment for errors across the care continuum [6]. To address these challenges, nurses must possess not only clinical knowledge but also patient safety competencies to identify hazards, respond to early warning signs, and promote a culture of safety [7].

The Canadian Patient Safety Institute outlines six core domains guiding safe nursing practice. The first domain, contributing to a culture of safety, promotes open communication and non-punitive error reporting, fostering a safer environment [8]. Working in teams emphasizes collaboration with healthcare professionals for coordinated care, while effective communication highlights the importance of clarity to prevent errors [9]. Managing safety risks involves identifying and mitigating hazards, while optimizing human and environmental factors focuses on balancing workloads and ergonomics to minimize fatigue-related errors [10]. The final domain, addressing adverse events, stresses early recognition, swift intervention, and transparent reporting, collectively empowering nurses to enhance patient safety [11].

Studies on patient safety competencies emphasize three critical dimensions: knowledge, attitudes, and skills, which are essential for navigating modern healthcare complexities [12]. Knowledge is foundational, encompassing the causes of errors, human factors, safety culture, risk management, and evidence-based safety practices. This understanding enables nurses to prevent errors and mitigate risks effectively. Research indicates that nurses proficient in these areas are better equipped to identify hazards and implement safety measures in clinical settings [13].

Equally important is the attitude nurses hold toward patient safety. A nurse’s perception of safety as a core part of their professional role directly influences their willingness to engage in safety practices [14]. Nurses who prioritize safety are more likely to take steps to prevent harm, such as double-checking medications, reporting near misses, and adhering to infection prevention protocols. A positive safety culture in healthcare organizations is vital for shaping nurses’ attitudes, encouraging them to adopt behaviors that minimize errors and enhance patient safety [15].

Finally, skills are essential for the practical application of patient safety knowledge. Nurses must be proficient in critical areas such as teamwork, communication, and clinical decision-making [16]. These skills are vital in recognizing signs of patient deterioration, safely administering medications, and using medical equipment correctly [17]. Effective communication is particularly crucial, as breakdowns in communication often lead to errors. Studies have consistently shown that nurses with strong teamwork and communication skills are more capable of preventing adverse events, emphasizing the importance of continuous skill development to enhance patient safety [18, 19].

Research indicates that while most nurses acknowledge the importance of patient safety, there are gaps in their formal education and training [7]. Habibi Soola et al. (2020) revealed that many nurses lack sufficient knowledge of safety standards, proper risk assessment methods, and the significance of systems-based practices [20]. Similarly, attitudes toward patient safety are often shaped by institutional culture [12]. In some settings, a punitive culture may discourage error reporting, while in others, a lack of leadership commitment may hinder safety efforts [21].

Several strategies have been proposed to enhance patient safety competencies among nurses, focusing on fostering a culture of safety, continuous education, and system improvements [22]. One vital strategy is system thinking, which involves understanding how components within a healthcare system interact and influence one another [5]. This approach encourages nurses to see the bigger picture, recognize patterns, and anticipate the consequences of decisions within a complex system [23]. Unlike the traditional, linear approach to problem-solving, system thinking addresses the intricate interdependencies within healthcare settings, leading to better safety outcomes [24]. It helps nurses consider how decisions at the individual or departmental level might affect other parts of the system, identify root causes of errors, streamline processes, and foster a culture of safety that transcends individual responsibility [25].

System thinking is a powerful approach to improving patient safety, encompassing several key dimensions. It promotes a holistic perspective, encouraging nurses to view healthcare as an interconnected system, enabling the identification of patterns and relationships to solve safety challenges [26]. It emphasizes root cause analysis, focusing on underlying factors of errors for long-term solutions. Interdisciplinary collaboration is crucial, fostering cooperation among healthcare professionals to develop comprehensive safety solutions [27]. Additionally, system thinking advocates for continuous learning and improvement, encouraging nurses to reflect on their practice, learn from mistakes, and make necessary changes. This mindset is vital in creating a culture of safety and adaptability in healthcare [28].

Nurses, due to their direct patient care role, are ideally positioned to benefit from system thinking [5]. They act as the final checkpoint for medication, monitor early signs of deterioration, and coordinate care. System thinking helps them foresee communication breakdowns, workflow inefficiencies, and minor errors that could lead to significant harm [29]. For example, recognizing a delay in lab results that could delay critical medication administration allows nurses to intervene proactively, improving patient safety [30].

Empirical evidence supports the positive impact of system thinking on patient safety. A study by Watts et al. (2024) found that nurses trained in system thinking demonstrated improved decision-making abilities and were more adept at recognizing system-level risks [29]. Mahsoon (2019) indicated that system thinking training led to better interdisciplinary collaboration, which reduced adverse events such as medication errors and patient falls [30]. Furthermore, organizations that implement system thinking report lower rates of complications and improved patient outcomes [31].

Despite the global emphasis on patient safety and system thinking, there remains a significant research gap in understanding the specific competencies required for nurses in developing countries, including Egypt. In Egypt, healthcare systems face unique challenges, such as limited resources, regulatory constraints, and high patient-to-nurse ratios. These factors make it difficult to implement comprehensive patient safety strategies [31]. While some studies have explored patient safety competencies in Egyptian hospitals [32, 33], there is limited research on how system thinking could be integrated into existing frameworks to enhance these competencies. The current healthcare landscape in Egypt, with its reliance on hierarchical decision-making and fragmented communication channels, often hinders the development of a safety culture [34]. Therefore, it is crucial to investigate how system thinking could be applied in this context to promote a safer, more adaptive healthcare environment.

This study aims to fill this gap by examining how system thinking influences patient safety competencies among nurses in Egypt. By addressing this research gap, the study seeks to provide actionable insights for healthcare managers and policymakers to design interventions that foster both individual competency development and system-wide improvements.

## Objectives of the study

This study has two objectives. First, to assess the levels of system thinking and patient safety competencies among nurses. Second, to explore the impact of system thinking on nurses’ patient safety competencies.

### Method

#### Research design

A cross-sectional exploratory research design was used to carry out this study. We used this design in our study as it allows for the simultaneous examination of system thinking and patient safety competencies among nurses at a specific point in time. This approach enables the identification of correlations between variables and facilitates insights into current practices without the need for longitudinal data collection.

#### Setting

This study was conducted in all 10 critical care units (ICUs) of Alexandria Main University Hospital, which is affiliated with Alexandria University, Egypt. The hospital provides public, non-paid healthcare services and has a total bed capacity of over 6,760 beds, with 1,126 beds allocated to the 10 ICUs. It is the largest educational university hospital in Alexandria and the first university hospital to make significant progress in meeting the requirements of the General Authority for Health Accreditation and Regulation (GAHAR) regarding patient safety.

#### Subjects

A sample of 289 nurses was selected using a convenience sampling method from a total population of 380 nurses in the study setting. The survey was distributed to all 380 eligible nurses, and 289 nurses participated, achieving the sample size required per G*Power analysis. Using G*Power (version 3.1.9.7) for linear multiple regression, the sample size was calculated to achieve a small effect size of 0.05 for F-test, a power of 85%, and a type 1 error of 0.05. Inclusion criteria required participants to have at least six months of experience providing direct or indirect care, ensuring familiarity with hospital policies, roles, and regulations. Participation was voluntary, and only those willing to take part were included.

## Study tools

Two tools were used in this study as follows:

### Tool (I): systems thinking scale (STS)

This tool was developed by Dolansky et al., (2020) [23] to assess system thinking in terms of nurses’ ability to recognize, understand and synthesize the sequence of events, causal sequences, possible multiple causes, different types of events, feedback, and interrelated factors. It consists of 20 items that use a 5-point Likert scale ranging from 1 = never to 5 = most of the time. A total score was computed by summing up the responses for each item. Scores can range from 20 to 100. This scale was categorized as the following: scores range from 20 < 46 low level of perceived system thinking, 46 < 73 moderate level of perceived system thinking, 73–100 high level of perceived system thinking. The reliability of tool was tested by Dolansky et al., (2020) and results revealed high reliability as Cronbach’s alpha coefficient was 0.89 [23].

### Tool (II): patient safety competency self-evaluation questionnaire (PSCSE)

This tool was developed by Lee et al., (2014) [35] to assess the level of patient safety competency among nurses. This questionnaire has 41 items in three dimensions: knowledge and awareness (6 items), skills (21 items), and attitudes (14 items). Concerning knowledge and awareness dimension, it is subdivided into two subcategories namely, patient safety culture (4 items), and error and cause analysis (2 items). Items of knowledge and awareness dimension were rated using a 5-point Likert scale ranging from 1 = not knowledgeable to 5 = very knowledgeable.

Regarding the skill dimension, it is subdivided into six subcategories: error reporting and response to an error (4 items), communication related to error (3 items), resource utilization/evidence-based practice (3 items), safe nursing practice (5 items), infection prevention (4 items), and precise communications during handoffs (2 items). Items were scored using a 5-point Likert scale, ranging from 1 = very uncomfortable to 5 = very comfortable. Finally, the attitude dimension is further subdivided into 4 subcategories: patient safety promotion/prevention strategy (4 items), responsibility of healthcare professionals for patient safety culture (4 items), error reporting and disclosing (4 items), and the components of patient safety culture (2 items). Items of the attitude dimension were assessed using a 5-point Likert scale, ranging from 1 = strongly disagree to 5 = strongly agree. The total score of the PSCSE was calculated by summing the scores for all three domains. Nurses’ perceived level of patient safety competency was categorized as follows: scores ranging from 41 to 95 indicate low level, 95–150 indicate moderate level, and 150–205 indicate high level. This tool has high reliability, with a Cronbach’s Alpha Coefficient score of 0.92 [17], and in the current study, it was 0.97.

In addition, personal and work-related characteristics data sheet was developed by the researcher and consist of questions related to nurses` age, gender, marital status, education level, years of experience in nursing, years of experience in current working unit, job title, and current working unit, and previous attendance of workshops related to patient safety and system thinking.

## Tools adaptation, validity and reliability

### Tools translation

The tools were translated from English into Arabic to adapt to the Egyptian culture then back-to-back translation was done. Two translators with a degree in translation and previous experience in written or oral translation in the field of healthcare and nursing were selected to translate the tool from English into Arabic. Each one was asked to translate the tool separately and independently. Both completed versions were unified, creating a single translated version of the questionnaires from English into Arabic. Then, two different bilingual translators with experience in healthcare and nursing related translation received the Arabic unified translated version and performed the back-translation of the tool from Arabic into English, resulting in a single back-translation. After both language versions became available, each item was checked to identify any inconsistencies and, wherever inconsistencies were found, the items were modified to stay as closely as possible to the original version in English. The final version of the questionnaires was revised by two members of the research team.

### Content validity

The tools were tested for their face and content validity by five experts in the field of the study. They were three professors and two assistant professors from the nursing administration department, Faculty of Nursing, Alexandria University. The panel of experts highlighted errors in punctuation, typography, and word choice. Based on their suggestions, certain terms were changed, and the tools were put in their final configuration. A pilot study was carried out on a number equal to 10% of nurses (*N* = 29) from Alexandria Main University hospital. It is intended to check and ensure the clarity of the tools, their applicability and feasibility, identify obstacles and problems that might be encountered during data collection, and establish the time required to complete the study questionnaire. The pilot study sample was excluded from the final research sample to maintain the integrity of the study results.

### Construct validity

Confirmatory factor analysis (CFA) was conducted using structural equation modeling (SEM) to assess the construct validity of the translated tools. The CFA evaluated the factor structure of the PSCSE and STS to ensure the tools have the same attributes after adaptation. Model fit was judged based on the following criteria: a root mean square error of approximation (RMSEA) ≤ 0.06 for a close fit, with values up to 0.08 considered acceptable [36, 37]. A goodness-of-fit index (GFI) of ≥ 0.90 was deemed adequate, though higher cut-offs (≥ 0.95) are recommended for studies with low factor loadings or smaller sample sizes [38]. Additionally, the comparative fit index (CFI) and incremental fit index (IFI) should exceed 0.95 for strong model support [39]. Based on these criteria, the CFA for the PSCSE questionnaire demonstrated good model fit (CFI = 1.000, IFI = 1.000, RMSEA = 0.049, X² = 3.673, *P* < 0.001). Similarly, the CFA for the STS indicated strong model fit (CFI = 1.000, IFI = 1.000, RMSEA = 0.055, X² = 4.070, *P* < 0.001), *see supplementary*.

### Reliability analysis

The reliability of the assessment tools was assessed using corrected item-total correlations, inter-dimension correlations, and internal consistency. Corrected item-total correlations evaluate the relationship between each item and the total score, excluding that item. In the PSCSE questionnaire, all items showed significant positive correlations with their respective sections and the overall score, ranging from 0.330 to 0.813. The STS also displayed significant item correlations, ranging from 0.503 to 0.717. Inter-dimension correlations examined the relationships among the dimensions within each tool and their overall scores, revealing positive and statistically significant correlations across all dimensions of both the PSCSE and STS, which underscores their interconnected nature. Internal consistency was evaluated using Cronbach’s alpha, a metric that indicates how reliably a set of items measures a single construct, with values from 0 to 1—higher values indicating better reliability. The PSCSE questionnaire’s Cronbach’s alpha ranged from 0.809 to 0.938 for its subscales, achieving an overall score of 0.956. The STS demonstrated values from 0.911 to 0.916, with an overall score of 0.917, indicating robust internal consistency for both instruments.

### Data collection

Data collection was conducted using hand-delivered questionnaires distributed to staff nurses across various categories during morning, evening, and night shifts. Each nurse was provided with an individualized briefing lasting approximately 10 min, during which the purpose of the study was explained, and general instructions on how to complete the questionnaires were provided. Specific instructions relevant to each questionnaire were also clarified. Nurses were encouraged to read each statement carefully and select the response option that best reflected their views. Completing the questionnaires took about 15 to 20 min for each nurse. Afterward, they were instructed to return the completed questionnaires directly to the researcher. The data collection process spanned two months, from early March 2024 to the end of April 2024.

### Ethical considerations

An official approval for conducting the study was obtained from the Research Ethics Committee (IRB00013620, SN: AU-20-8-78), Faculty of Nursing, Alexandria University, Egypt. Official permission for conducting the study was obtained from the hospital administrators to collect the necessary data. The study was conducted in accordance with the requirements of the Helsinki Declaration. A written informed consent from nurses was obtained after providing an appropriate explanation about the aim of the study. The confidentiality of the data was maintained. The anonymity of the study subjects was maintained. Subjects participated in the study voluntarily and had the right to withdraw at any time from the study without any consequences.

### Statistical analysis

Data analysis was conducted using SPSS version 25, ensuring all entered data were checked for errors. Descriptive statistics included the number of entries, minimum and maximum values, arithmetic mean, and standard deviation. Categorical variables were summarized using frequency and percentage. Inferential statistics were employed to assess the relationships between the variables of interest. The inter-correlation of research variables was analyzed using Pearson’s correlation coefficient (r). A correlation is considered perfect if r equals 1 or -1, strong if r is less than − 0.5 or greater than 0.5, moderate if r falls between 0.3 and 0.5 or between − 0.5 and − 0.3, weak if r is less than 0.3 or greater than − 0.3, and indicates no association if r equals 0. The significance of the correlation coefficient was evaluated at *p* ≤ 0.05. Additionally, hierarchical regression analysis was conducted to explore the degree of variation in patient safety competencies among nurses as influenced by system thinking while controlling for demographic factors.

## Results

Table 1 provides demographic and professional details of 289 nurses in the study. The largest proportion of nurses (56.1%) were aged between 20 and less than 30 years, with a mean age of 30.15 ± 8.1 years. Most nurses (45.3%) held a bachelor’s degree, and only 0.3% had a master’s degree. Most nurses (45.3%) held a bachelor’s degree, and only 0.3% had a master’s degree. Regarding experience, 60.9% had 1–10 years of work experience, with an average of 8.07 ± 7.69 years. Similarly, 60.2% had worked in their current position for 1–10 years. More than half (53.6%) were single. Notably, 65.1% did not attend any patient safety courses, while 34.9% did, and 17% found them somewhat beneficial. Additionally, 64% did not attend patient safety workshops, while 36% did, with 22.8% finding them highly beneficial.

Table 2 provides an overview of nurses’ competencies in systems thinking and patient safety. The mean score for systems thinking is 82.36 ± 12.14, indicating moderate to high proficiency. The overall mean score for patient safety competencies (PSC) is 162.74 ± 23.56, which, according to the study’s scoring criteria, reflects a high level of competency. However, variability exists across domains. The knowledge/awareness domain shows adequate awareness (21.03 ± 4.79), while error & cause analysis has a lower score (7.24 ± 1.92), indicating a need for improvement. Skills related to safe practice fall within the high range (85.08 ± 13.17), particularly in infection prevention and safe nursing practice. However, precise communication during hand-offs scores lower (8.71 ± 1.74), signaling gaps. Attitudes toward patient safety are moderate (56.64 ± 8.11), with error reporting & disclosing scoring 14.69 ± 2.81. Lower scores in patient safety culture (8.77 ± 1.56) and safety promotion strategies (16.18 ± 2.81) suggest areas for strengthening a proactive safety environment.

Table 3 Shows a strong positive correlation (*r* = 0.605, *p* ≤ 0.05) between systems thinking and total patient safety competencies (PSCSE) among nurses, indicating that higher systems thinking abilities are linked to better patient safety outcomes. Subdomains of knowledge (*r* = 0.489), skills (*r* = 0.568), and attitudes (*r* = 0.546) also moderately correlate with systems thinking, suggesting that nurses with strong systems thinking are better equipped with essential safety skills. Moderate correlations are found between systems thinking and error & cause analysis (*r* = 0.449), error reporting & response (*r* = 0.511), and communication during hand-offs (*r* = 0.532), emphasizing the role of systems thinking in effective teamwork and error prevention. Additionally, there are strong correlations between years of experience, qualifications, and both systems thinking and safety competencies.

**Table 1:** Personal and professional characteristics of the studied nurses

Items		Total (*n* = 289)	
		N	%
Age Group	20–29	162	56.1%
	30–39	90	31.1%
	40–49	27	9.3%
	50+	10	3.5%
	Mean ± S.D.	30.15 ± 8.11	
Gender	Males	89	31%
	Females	200	69%
Educational Qualification	Diploma degree of nursing	79	27.3%
	Associated degree of nursing	78	27.0%
	Bachelor’s degree of nursing	131	45.3%
	Master’s degree of nursing	1	0.3%
Experience in the Nursing Profession (year)	< 1	16	5.5%
	1–9	176	60.9%
	10–19	61	21.1%
	20–29	28	9.7%
	30+	8	2.8%
	Mean ± S.D.	8.07 ± 7.69	
Experience in the Current working unit (year)	< 1	62	21.5%
	1–9	174	60.2%
	10–19	31	10.7%
	20–29	20	6.9%
	30+	2	0.7%
	Mean ± S.D.	5.21 ± 6.37	
Marital Status	Single	155	53.6%
	Married	119	41.2%
	Widowed	7	2.4%
	Divorced	8	2.8%
Previous attendance of Patient Safety Courses	No	188	65.1%
	Yes	101	34.9%
Usefulness of Patient Safety Courses	No benefit at all	1	0.3%
	Benefited to some extent	49	17.0%
	Very beneficial	43	14.9%
Previous attendance of patient safety workshops	No	185	64.0%
	Yes	104	36.0%
Usefulness of workshops	No benefit at all	26	9.0%
	Benefited to some extent	26	9.0%
	Very beneficial	66	22.8%

**Table 2:** Descriptive statistics of study variables among nurses

Item	Total (*n* = 289)	
	Mean	± S.D.
**Systems Thinking**	**82.36**	**12.14**
**Patient safety competency Questionnaire**	**162.74**	**23.56**
**Knowledge/Awareness**	**21.03**	**4.79**
Patient Safety Culture	13.79	3.24
Error & Cause Analysis	7.24	1.92
**Skills**	**85.08**	**13.17**
Error Reporting & Response to an Error	15.22	3.51
Communication Related to Error	11.37	2.19
Resource Utilization/Evidence-Based Practice	10.11	2.23
Safe Nursing Practice	21.84	3.70
Infection Prevention	17.83	2.99
Precise Communications During Hand-Offs	8.71	1.74
**Attitudes**	**56.64**	**8.11**
Patient Safety Promotion/Prevention Strategy	16.18	2.81
Responsibility of Health Care Professionals for Patient Safety Culture	17.01	2.68
Error Reporting & Disclosing	14.69	2.81
Components of Patient Safety Culture	8.77	1.56

**Table 3:** Pearson Correlation matrix between systems thinking and patient safety competencies among the studied nurses

Parameter/ Variable	Systems Thinking Scale	PSCSE	PSCSE Knowledge	Patient Safety Culture	Error & Cause Analysis	PSCSE Skills	Error Reporting & Response to an Error	Communication Related to Error	Resource Utilization/Evidence-Based Practice	Safe Nursing Practice	Infection Prevention	Precise Communications During Hand-Offs	PSCSE Attitudes	Patient Safety Promotion/Prevention Strategy	Responsibility of Health Care Professionals for Patient Safety Culture	Error Reporting & Disclosing	Components of Patient Safety Culture
Gender	0.225*	0.100	0.023	0.047	-0.023	0.097	0.106	0.040	-0.063	0.082	0.140*	0.137*	0.119*	0.034	0.114	0.091	0.196*
Age	0.254^*^	0.406^*^	0.353^*^	0.332^*^	0.321^*^	0.386^*^	0.272^*^	0.310^*^	0.305^*^	0.340^*^	0.304^*^	0.343^*^	0.346^*^	0.341^*^	0.308^*^	0.213^*^	0.273^*^
Qualification	0.763*	0.633*	0.545*	0.502*	0.514*	0.575*	0.532*	0.454*	0.270*	0.456*	0.483*	0.560*	0.582*	0.484*	0.508*	0.433*	0.499*
Years of experience	0.231^*^	0.433^*^	0.363^*^	0.343^*^	0.328^*^	0.400^*^	0.288^*^	0.314^*^	0.308^*^	0.358^*^	0.322^*^	0.344^*^	0.394^*^	0.352^*^	0.349^*^	0.285^*^	0.302^*^
Years of experience in the current unit	0.260^*^	0.478^*^	0.429^*^	0.415^*^	0.372^*^	0.450^*^	0.368^*^	0.359^*^	0.324^*^	0.384^*^	0.351^*^	0.374^*^	0.405^*^	0.332^*^	0.341^*^	0.329^*^	0.327^*^
Systems Thinking Scale		0.605*	0.489*	0.457*	0.449*	0.568*	0.511*	0.491*	0.281*	0.453*	0.463*	0.532*	0.546*	0.463*	0.487*	0.388*	0.464*
PSCSE			0.813*	0.779*	0.717*	0.956*	0.794*	0.734*	0.598*	0.843*	0.786*	0.800*	0.872*	0.731*	0.756*	0.656*	0.732*
PSCSE Knowledge				0.959*	0.879*	0.724*	0.654*	0.625*	0.471*	0.613*	0.506*	0.597*	0.596*	0.503*	0.464*	0.491*	0.509*
Patient Safety Culture					0.708*	0.699*	0.621*	0.589*	0.493*	0.599*	0.477*	0.567*	0.561*	0.496*	0.439*	0.436*	0.484*
Error & Cause Analysis						0.629*	0.584*	0.566*	0.344*	0.521*	0.457*	0.533*	0.541*	0.420*	0.420*	0.489*	0.454*
PSCSE Skills							0.808*	0.754*	0.618*	0.891*	0.846*	0.846*	0.727*	0.624*	0.637*	0.516*	0.629*
Error Reporting & Response to an Error								0.664*	0.391*	0.599*	0.530*	0.577*	0.609*	0.451*	0.511*	0.498*	0.574*
Communication Related to Error									0.471*	0.555*	0.441*	0.564*	0.539*	0.456*	0.456*	0.415*	0.446*
Resource Utilization/Evidence-Based Practice										0.432*	0.378*	0.447*	0.457*	0.510*	0.339*	0.335*	0.269*
Safe Nursing Practice											0.827*	0.732*	0.640*	0.536*	0.597*	0.417*	0.585*
Infection Prevention												0.813*	0.610*	0.531*	0.565*	0.384*	0.551*
Precise Communications During Hand-Offs													0.599*	0.532*	0.540*	0.399*	0.507*
PSCSE Attitudes														0.815*	0.886*	0.778*	0.804*
Patient Safety Promotion/Prevention Strategy															0.657*	0.404*	0.573*
Responsibility of Health Care Professionals for Patient Safety Culture																0.567*	0.682*
Error Reporting & Disclosing																	0.539*

*: Statistically significant at *p* ≤ 0.05

Table 4 presents a hierarchical multiple linear regression analysis that explores factors affecting patient safety competencies among the studied nurses. In Model 1, gender, age, qualifications, years of experience, and years of experience in the current unit were evaluated. The results indicate that age (B = 0.631, *p* < 0.001), qualifications (B = 4.739, *p* < 0.001), years of experience (B = 2.621, *p* < 0.001), and years of experience in the current unit (B = 1.529, *p* < 0.001) are significant predictors of patient safety competencies. However, gender was not a significant factor in predicting competencies (*p* = 0.704). The overall model explained 58.8% of the variance in patient safety competencies (R² = 0.588, F = 80.697, *p* < 0.001).

In Model 2, the Systems Thinking Scale was added, which significantly improved the model. Systems thinking emerged as a strong predictor of patient safety competencies (B = 0.663, *p* < 0.001), indicating that nurses who scored higher in systems thinking demonstrated higher patient safety competencies. The inclusion of systems thinking increased the explained variance to 67.7% (R² = 0.677, F = 98.647, *p* < 0.001). Age, qualifications, years of experience, and years of experience in the current unit remained significant predictors, though their beta values slightly decreased, suggesting that systems thinking partially mediates their effect. These results emphasize the critical role of systems thinking in enhancing patient safety competencies among nurses.

**Table 4:** Hierarchical Multiple Linear Regression Analysis to assess factors affecting patient safety competencies of the studied nurses

	Step 1 (Model 1)					Step 2 (Model 2)					
	B	S. E	β	t	*p*	B	S. E	β	t	*p*	
**(Constant)**	121.478	5.367		22.636*	< 0.001*	82.094	6.515		12.601*	< 0.001*	
Gender	-0.775	2.039	-0.015	-0.380	0.704	-3.253	1.829	-0.064	-1.779	0.076	
Age	0.631	0.163	0.217	3.863*	< 0.001*	0.434	0.146	0.149	2.964*	0.003*	
Qualification	4.739	1.309	0.169	3.620*	< 0.001*	2.945	1.178	0.105	2.501*	0.013*	
Years of experience	2.621	0.332	0.855	7.905*	< 0.001*	2.313	0.296	0.755	7.815*	< 0.001*	
Years of experience in the current unit	1.529	0.371	0.414	4.123*	< 0.001*	1.273	0.330	0.344	3.858*	< 0.001*	
**Systems Thinking Scale**						0.663	0.075	0.342	8.846*	< 0.001*	
	*R* = 0.767,** R**^**2**^ **= 0.588**, F = 80.697^*^, *p* < 0.001^*^					*R* = 0.823,** R**^**2**^ **= 0.677**, F = 98.647^*^, *p* < 0.001^*^					
**R**^**2**^**change = 0.670**, F = 98.647^*^, *p* < 0.001^*^											

**B**: Unstandardized Coefficients, **β**: Standardized Coefficients, t: t-test of significance, **R**^**2**^: Coefficient of determination, **SE**: standard error, t: t-test of significance *: p is statistically significant at the 0.05 (2-tailed)

## Discussion

The hallmark of contemporary nursing practice is building different nursing competencies that help nurses stay diligent in the face of eminent threats and challenges [40]. The current study aims to examine the level of patient safety competencies among nurses and test the effect of system thinking on shaping these competencies. The findings highlight that system thinking is a powerful determinant and predictor of patient safety competencies among nurses.

### Patient safety competencies among nurses

Interestingly, the current study revealed that nurses possess high levels of patient safety competencies. This finding is significant as it underscores the crucial role that nurses play in ensuring patient safety within healthcare settings. The competencies encompass a broad range of skills and knowledge areas, including infection prevention, medication management, and the ability to identify and mitigate potential safety risks. The high level of competency observed among nurses is a testament to the rigorous training and continuous professional development they undergo, emphasizing the importance of well-educated and skilled nursing staff in maintaining and enhancing patient safety standards.

Our study explored dimensions of patient safety competencies among nurses highlighted several key skills, including infection prevention, error reporting, and precise communication, along with knowledge and attitudes towards patient safety. The results revealed that nurses exhibited moderate to high levels of competence across these dimensions, with particularly high proficiency in the skill dimension. This finding underscores the critical role of practical skills in ensuring patient safety and the effectiveness of nurses in maintaining high standards of care within healthcare settings.

The high level of patient safety competencies among nurses can be attributed to the emphasis on safety in nursing education and professional standards, including training in safety principles, risk management, and evidence-based practices [41]. Continuous professional development, such as workshops and certifications, keeps nurses updated on safety protocols. Additionally, the collaborative nature of healthcare teams promotes shared learning, best practices, and collective efforts to enhance patient outcomes [42].

In Egypt, high patient safety competencies among nurses are driven by several factors. The healthcare sector has been undergoing reforms aimed at improving care quality and patient safety, including the establishment of accreditation bodies and the implementation of quality standards in hospitals [4]. Additionally, Egyptian nurses often work under challenging conditions, such as high patient-to-nurse ratios and limited resources, which necessitate a higher level of competence and resourcefulness to ensure patient safety despite these constraints [5].

Supporting studies confirm the high levels of patient safety competencies among nurses. Okuyama et al. (2011) found consistent evidence of moderate to high competency levels across healthcare settings and specialties [1]. Alreshidi et al. (2021) revealed strengths in error reporting and communication in a simulation setting [43]. Karanikas et al. (2022) also found nurses across various regions displaying satisfactory level of patient safety competencies [44]. Additionally, a comparative study by Fu et al. (2022) showed nurses outperforming other healthcare professionals, emphasizing their training and experience in patient safety [41].

Despite these positive findings, the error and cause analysis subdomain showed relatively lower scores compared to other patient safety competencies in our study. This highlights a gap in nurses’ abilities to analyze errors and prevent recurrence. Studies by Son et al. (2019) [11] and Park et al. (2024) [12] revealed that error analysis and reporting often lag due to institutional barriers, such as fear of blame and limited resources for investigations. Kalsoom et al. (2023) [16] found that while nurses generally understand patient safety principles, significant gaps remain in error reporting and safety protocol use. These discrepancies may stem from differences in training programs, organizational cultures, and resource availability. The findings suggest that while many nurses demonstrate strong patient safety competencies, others require additional education and support to address these specific gaps effectively.

Our findings revealed significant correlations between patient safety competencies and background variables like age, qualifications, and years of experience. Nurses who were older, more experienced, and held higher qualifications demonstrated higher levels of patient safety competencies compared to their peers. This aligns with studies emphasizing the role of education and experience in enhancing patient safety [40]. Experienced nurses may better understand safety principles, improving competencies in error reporting, communication, and infection prevention. Studies by Yan et al. (2021) [9] and Tai et al. (2024) [10] similarly show that higher qualifications and experience enhance safety practices, including root cause analysis and evidence-based care.

### System thinking among nurses

System thinking scores among the nurses indicate a moderately high level of systems thinking, with a significant correlation between systems thinking and variables such as age, qualifications, and years of experience. These results suggest that systems thinking is more developed among older, more experienced nurses with higher qualifications, which aligns with the findings related to patient safety competencies.

This finding could be attributed to the complex and under-resourced healthcare system in Egypt, which necessitates that nurses develop strong systems thinking skills to navigate and manage systemic challenges efficiently [6]. This environment encourages a holistic and strategic approach to problem-solving. Nurses’ daily responsibilities, which involve coordinating care across various departments, naturally foster a broader systemic perspective [32]. The increasing use of technology and data analytics further enhances their ability to understand systemic patterns and relationships [5].

In the same line, research indicates that nurses possess moderate to high levels of systems thinking, which significantly benefits patient outcomes. Ahmed and Ibrahim (2023) found that many nurses in Egypt recognize the importance of understanding the broader healthcare system [25]. Additionally, Kakemam et al. (2022) revealed that nurses with strong system thinking skills are better at identifying root causes of patient care issues, leading to more effective interventions and improved patient safety [17]. Moazez et al. (2020) highlighted that these nurses excel in coordinating care across various disciplines, essential for managing complex health issues [45].

On the other hand, some studies question its extent and practical application in daily practice. Some studies question the extent and practical application of systems thinking in daily nursing practice, citing barriers like heavy workloads, insufficient training, and organizational constraints [33]. The demanding nature of nursing often leaves little time for reflective practice, hindering the use of systems thinking principles [5]. Cho and Baek (2020) noted that these realities can obstruct consistent use of these skills [24]. This view highlights the need for targeted, context-specific research to understand its effective application in nursing practice.

### Role of system thinking toward patient safety competencies among nurses

Our results demonstrate the significant effect of systems thinking on patient safety competencies since when systems thinking was added in model 2 of regression analysis, the explained variance increased to 67.7%. This indicates that systems thinking significantly improves the predictive power of the model and plays a crucial role in enhancing patient safety competencies among nurses.

The strong positive correlation between systems thinking and several subdimensions of patient safety competencies in our study is noteworthy. For example, systems thinking is significantly correlated with knowledge, skills, and error reporting. These findings suggest that nurses who score higher on systems thinking are better able to apply their knowledge in practice, identify errors, and communicate safety concerns effectively. This aligns with the findings of Linnéusson et al. (2022) [46] and Khalil & Lakhani (2022) [47], who argued that systems thinking is essential for proactive risk identification and mitigation.

Manley et al. (2016) noted that systems thinking is closely linked to the knowledge component of safety competence [48]. Souda et al. (2024) demonstrated that incorporating systems thinking concepts into nursing education leads to a better understanding of the connections between individual actions and overall patient safety within the healthcare system [34]. Kakemam et al. (2022) confirmed that nurses' systems thinking significantly predicts their patient safety competency, highlighting the necessity for training opportunities, mentorship, and supportive leadership to enhance professionalism and systems thinking among nurses [17].

Interestingly, the effect of systems thinking on precise communication during hand-offs was particularly strong in this study​. This suggests that systems thinking not only improves individual competencies but also facilitates better teamwork and communication, which are critical for maintaining patient safety during transitions of care. This finding is consistent with Hwang and Park (2024) who reported that healthcare teams that adopt systems thinking are better able to coordinate care and prevent errors during hand-offs [49].

Our results also highlight how systems thinking impacts specific subdimensions of patient safety competencies. For example, the moderate correlation between systems thinking and infection prevention suggests that nurses who adopt a systems perspective are better able to anticipate and prevent healthcare-associated infections​. Infection prevention is a critical area of patient safety, and systems thinking helps nurses understand how factors such as hygiene, equipment sterilization, and patient flow interact to influence infection rates.

However, the relatively weaker correlation between systems thinking and safe nursing practice suggests that while systems thinking is important, it may not be sufficient on its own to ensure safe practices​. Other factors, such as workload, staffing levels, and organizational culture, may also play a role in shaping nurses’ ability to maintain safety at the bedside [50]. This finding is consistent with Chang and Tsai (2024) who clarified that while systems thinking improves error identification, it does not always translate to improved safety outcomes without the necessary organizational support [51].

## Implications of the study

This study has theoretical, practical, and broader implications.

### Theoretical implications

The study findings underscore the significant role of systems thinking in enhancing patient safety competencies among nurses. Theoretical models of nursing competency and patient safety should incorporate systems thinking as a critical component. This research adds to the growing body of literature that connects cognitive frameworks, such as systems thinking, to practical nursing outcomes like error prevention, effective communication, and safety culture promotion. It advances the theory that systems thinking is not just a supplementary skill but a foundational competency necessary for addressing the complexities of modern healthcare.

### Practical implications

Our study highlights important implications for nurses, nurse mangers, and healthcare organizations. For nurses, the results highlight the importance of developing systems thinking as part of their professional growth. Systems thinking skills can directly improve patient outcomes by enhancing the nurse’s ability to prevent errors, communicate effectively, and foster a culture of safety. Nurses should engage in continuous education and simulation-based training to strengthen their systems thinking capabilities, which can also help them navigate complex healthcare environments.

Nurse managers can play a pivotal role in fostering systems thinking among their staff by providing training programs that emphasize interdisciplinary collaboration, error analysis, and systems-based problem-solving. Managers should also promote an environment where reflective practice and team-based learning are encouraged. This will empower nurses to integrate systems thinking into their daily activities, which can help in identifying and addressing root causes of errors.

Healthcare organizations should invest in comprehensive training programs that promote systems thinking and patient safety competencies. Additionally, policies should be developed to integrate systems thinking into both organizational culture and clinical practice.

### Broader implications

Beyond the individual and organizational levels, systems thinking should be incorporated into nursing curricula and national patient safety standards. Educators and policymakers should develop standardized modules that include systems thinking to prepare future nurses for the complexities of healthcare. This could help mitigate the impact of external factors, such as resource constraints and workload, on the ability of nurses to provide safe care. In a broader context, strengthening systems thinking in healthcare may also facilitate innovations in policy, healthcare management, and technology integration.

## Limitations and Future Research directions

This study highlights the impact of systems thinking on patient safety but has limitations. Its cross-sectional design limits causal inference, suggesting future research should use longitudinal designs to explore systems thinking over time. The study’s specific geographic and organizational context limits generalizability, so extending research to diverse settings and countries is necessary. Additionally, while the study focuses on nurses, future research should examine the impact of systems thinking on patient safety among other healthcare professionals, such as doctors and allied health workers.

## Conclusion

This study demonstrates that systems thinking is a critical predictor of patient safety competencies among nurses. Enhancing nurses’ systems thinking abilities, they can significantly improve patient care quality and safety. The findings show that nurses with high systems thinking skills are better equipped to identify errors, foster effective communication, and implement safety strategies. This highlights the need for healthcare organizations to prioritize systems thinking in both training and practice. Incorporating systems thinking into nursing education, professional development, and healthcare policies can strengthen patient safety competencies across the board.

## Electronic supplementary material

Below is the link to the electronic supplementary material.

Supplementary Material 1

## Data Availability

The datasets and materials of the current study are available from the corresponding author on reasonable request.

## Abbreviations

CFA: Confirmatory Factor Analysis
SEM: Structural Equation Modeling
STS: System Thinking Scale
PSCSE: Patient Safety Competency Self-Evaluation
