# Supplementary material for: Exploring the hidden synergy between system thinking and patient safety competencies among critical care nurses: a cross-sectional study
Source: BMC Nurs. 2025 Jan 31;24:114. doi: 10.1186/s12912-025-02717-6 (PMC11783926; doi:10.1186/s12912-025-02717-6)
Supplement: Supplementary file 1 — Supplementary Material 1 [file 12912_2025_2717_MOESM1_ESM.docx]

| **Model fit parameters** | **Tool (I) : Systems Thinking Scale (STS)** | **Tool II: Patient Safety Competency Self-Evaluation Questionnaire (PSCSE)** |
| --- | --- | --- |
| **CFI** | 1.000 | 1.000 |
| **IFI** | 1.000 | 1.000 |
| **RMSEA** | 0.055 | 0.049 |
| **Model χ^2^** | 4.070 | 3.673 |
| **Significance** | <0.001* | <0.001* |


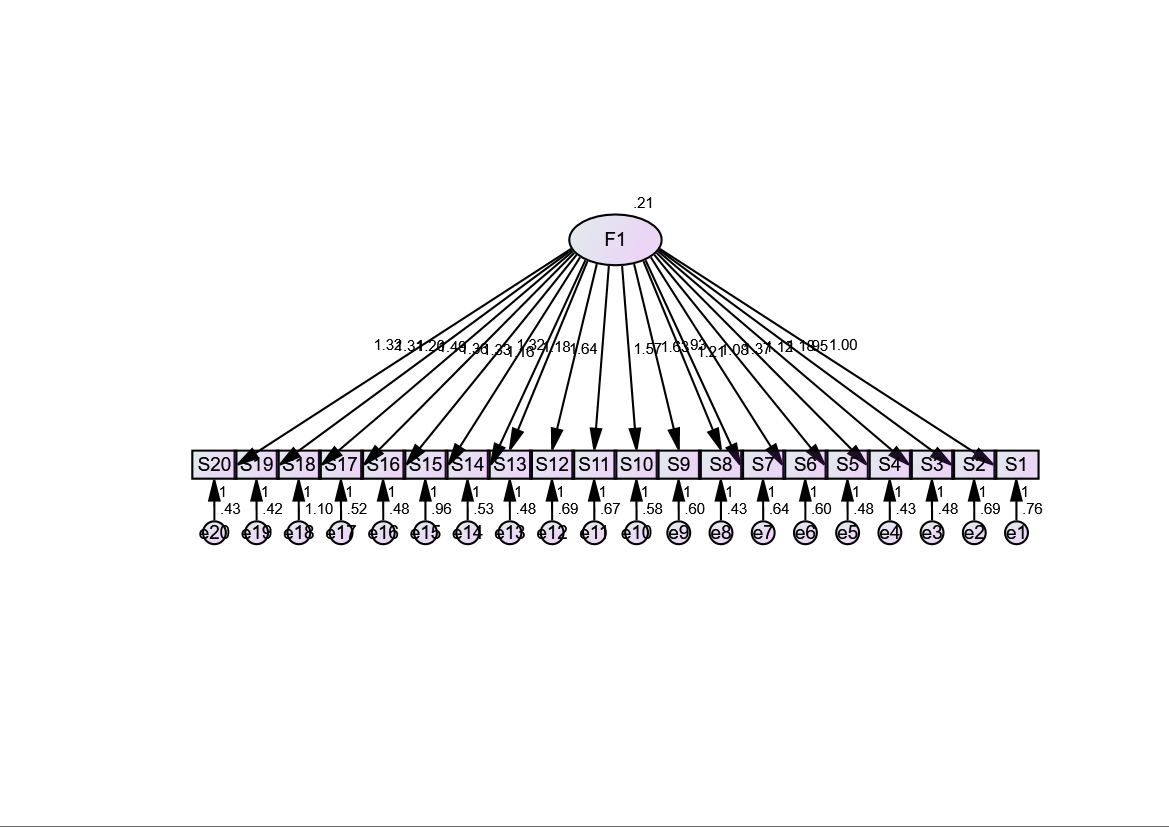


**Figure (S1): Confirmatory factor analysis (CFA) by Structure Equation Modeling (SEM) Tool (I): Systems Thinking Scale (STS)**


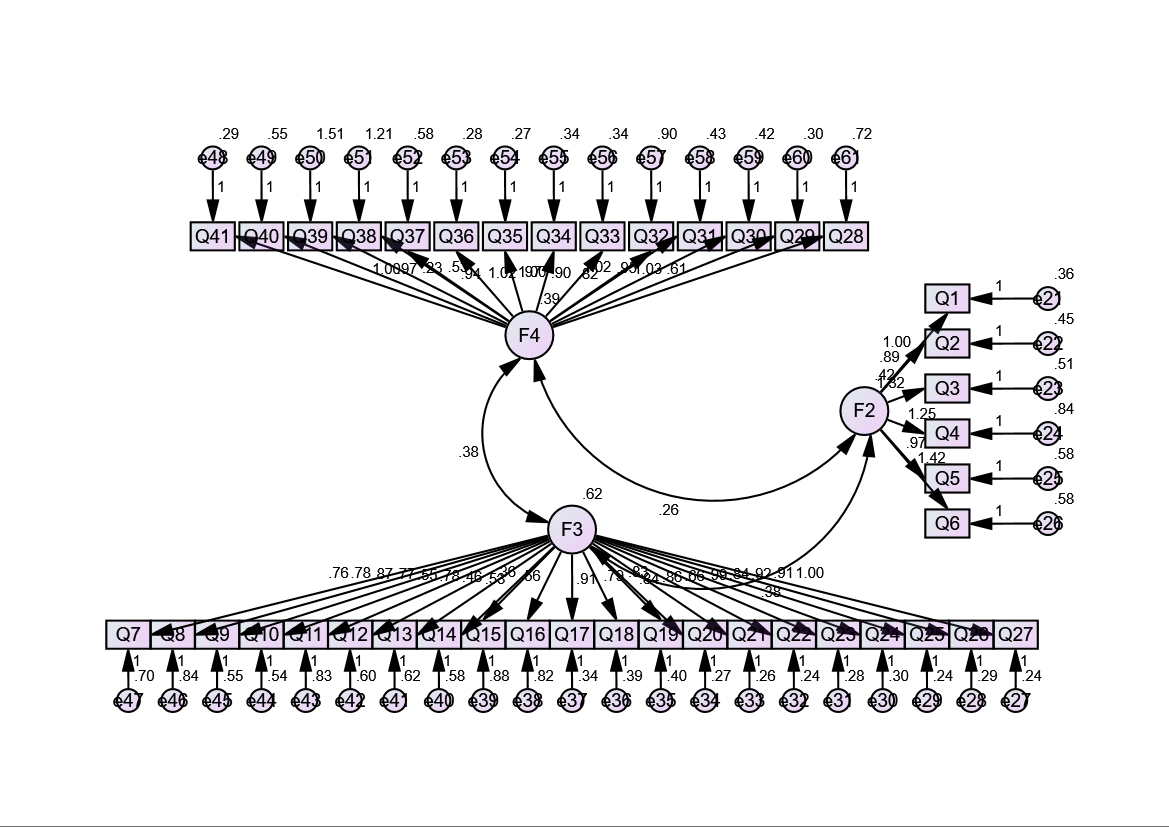


**Figure (S2): Confirmatory factor analysis (CFA) by Structure Equation Modeling (SEM) Tool II: Patient Safety Competency Self-Evaluation Questionnaire (PSCSE)**

**F2: Knowledge and awareness**

**F3: Skills**

**F4: Attitudes**
